# Prediction of appropriate implantable cardioverter-defibrillator therapy using machine learning and routinely available clinical data

**DOI:** 10.1093/ehjdh/ztag102

**Published:** 2026-06-26

**Authors:** Toshinori Chiba, Serafina Wegner, Emanuel Heil, Verena Tscholl, Robert Haettasch, Patrick Nagel, Johannes Lucas, Nikolaos Dagres, Felix Balzer, Alexander Meyer, Wilhelm Haverkamp, Florian Blaschke, Gerhard Hindricks, Felix Hohendanner

**Affiliations:** Department of Cardiology, Deutsches Herzzentrum der Charité, Charitéplatz 1, 10117 Berlin, Germany; Department of Cardiology, Deutsches Herzzentrum der Charité, Charitéplatz 1, 10117 Berlin, Germany; Department of Cardiology, Deutsches Herzzentrum der Charité, Charitéplatz 1, 10117 Berlin, Germany; DZHK (German Centre for Cardiovascular Research), partner Site Berlin, Berlin, Germany; Berlin Institute of Health, Charité—Universitätsmedizin Berlin, Anna-Louisa-Karsch-Straße 2, 10178 Berlin, Germany; Department of Cardiology, Deutsches Herzzentrum der Charité, Charitéplatz 1, 10117 Berlin, Germany; DZHK (German Centre for Cardiovascular Research), partner Site Berlin, Berlin, Germany; Department of Cardiology, Deutsches Herzzentrum der Charité, Charitéplatz 1, 10117 Berlin, Germany; DZHK (German Centre for Cardiovascular Research), partner Site Berlin, Berlin, Germany; Department of Cardiology, Deutsches Herzzentrum der Charité, Charitéplatz 1, 10117 Berlin, Germany; DZHK (German Centre for Cardiovascular Research), partner Site Berlin, Berlin, Germany; Department of Cardiology, Deutsches Herzzentrum der Charité, Charitéplatz 1, 10117 Berlin, Germany; DZHK (German Centre for Cardiovascular Research), partner Site Berlin, Berlin, Germany; Department of Cardiology, Deutsches Herzzentrum der Charité, Charitéplatz 1, 10117 Berlin, Germany; DZHK (German Centre for Cardiovascular Research), partner Site Berlin, Berlin, Germany; Institute of Medical Informatics, Charité—Universitätsmedizin Berlin, Corporate Member of Freie Universität Berlin and Humboldt-Universität zu Berlin, Invalidenstraße 90, 10115 Berlin, Germany; Institute of Artificial Intelligence in Medicine, Charité—Universitätsmedizin Berlin, Augustenburger Platz 1, 13353 Berlin, Germany; Department of Cardiology, Deutsches Herzzentrum der Charité, Charitéplatz 1, 10117 Berlin, Germany; DZHK (German Centre for Cardiovascular Research), partner Site Berlin, Berlin, Germany; Department of Cardiology, Deutsches Herzzentrum der Charité, Charitéplatz 1, 10117 Berlin, Germany; DZHK (German Centre for Cardiovascular Research), partner Site Berlin, Berlin, Germany; Department of Cardiology, Deutsches Herzzentrum der Charité, Charitéplatz 1, 10117 Berlin, Germany; DZHK (German Centre for Cardiovascular Research), partner Site Berlin, Berlin, Germany; Department of Cardiology, Deutsches Herzzentrum der Charité, Charitéplatz 1, 10117 Berlin, Germany; DZHK (German Centre for Cardiovascular Research), partner Site Berlin, Berlin, Germany; Berlin Institute of Health, Charité—Universitätsmedizin Berlin, Anna-Louisa-Karsch-Straße 2, 10178 Berlin, Germany

**Keywords:** Implantable cardioverter-defibrillator, Machine learning, Risk stratification, Ventricular arrhythmia, Non-sustained ventricular tachycardia

## Abstract

**Aims:**

Risk stratification for appropriate implantable cardioverter-defibrillator (ICD) therapy remains imprecise when based on conventional clinical variables alone. We aimed to develop and geographically validate a machine-learning model that integrates routinely available clinical, electrocardiogram, and device interrogation/programming parameters, and quantify the incremental value of non-sustained ventricular tachycardia (NSVT).

**Methods and results:**

We retrospectively analysed 514 ICD recipients implanted between 2020 and 2025 at two hospital sites (development cohort) and an independent cohort of 220 patients from a third site (external validation). The endpoint was appropriate ICD therapy (anti-tachycardia pacing or shock). Models were trained using nested stratified cross-validation in the development cohort, and the final refitted model was applied to the external cohort without recalibration. In the development cohort, 77/514 patients (15%) experienced appropriate ICD therapy (follow-up 404 days). A base model (histogram-based gradient boosting) excluding NSVT achieved modest discrimination [receiver operating characteristic (ROC)-area under the curve (AUC) 0.624; average precision 0.198]. Adding NSVT improved performance (ROC-AUC 0.805; average precision 0.432). Logistic regression with NSVT achieved comparable discrimination (ROC-AUC 0.78; average precision 0.43), indicating a largely NSVT-driven gain. External validation (event rate 51/220, 23%) confirmed good discrimination (ROC-AUC 0.815; average precision 0.583) with acceptable calibration (Brier score 0.137). At the pre-specified threshold of 0.16 in external validation, sensitivity was 0.78 and specificity was 0.74.

**Conclusion:**

A machine-learning model integrating routinely available clinical, electrocardiogram, and ICD programming/interrogation data may enable the prediction of appropriate ICD therapy with preserved performance on geographic external validation. NSVT was the dominant contributor to predictive performance across modelling approaches.

## Graphical Abstract

**Graphical Abstract:**
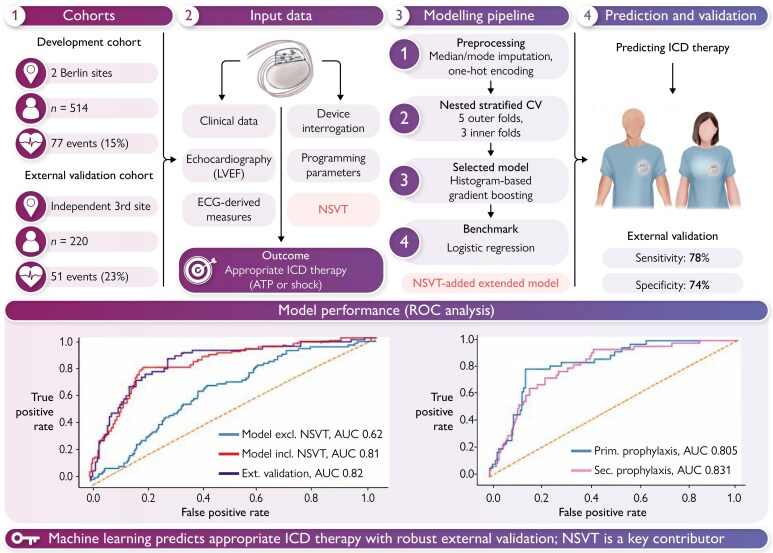

## Introduction

Implantable cardioverter-defibrillators (ICDs) are a cornerstone of sudden cardiac death prevention in patients at increased risk of malignant ventricular arrhythmias. While randomized trials have established mortality benefit in selected populations,^1–4^ a substantial proportion of ICD recipients do not experience appropriate device therapy over long-term follow-up, whereas others experience recurrent therapies with associated morbidity. Although the European Society of Cardiology (ESC) and the Heart Rhythm Society guidelines adopt a left ventricular ejection fraction (LVEF) < 35% as the criterion for primary prophylaxis ICD implantation.^5,6^ An analysis from the PROFID project suggested that LVEF showed limited ability to accurately predict individual sudden cardiac death risk in patients after myocardial infarction.^7^ A substantial proportion of ICD recipients never receive appropriate therapy and die from a non-arrhythmic cause, whereas others experience malignant ventricular arrhythmias despite apparently modest risk profiles.^8^ This imbalance underscores the limitations of conventional risk stratification strategies, which rely heavily on LVEF and broad clinical categorizations.

Contemporary ICD programming strategies (e.g. higher rate cut-offs, longer detection times, and multiple detection zones) and advances in heart failure therapy have reduced shock rates, but they have also increased heterogeneity of individual arrhythmic risk. As a consequence, conventional risk stratification based on a limited set of clinical covariates remains insufficient for individualized prediction of ICD therapy.

Machine-learning (ML) methods can integrate higher-dimensional clinical data with electrocardiogram (ECG) and device interrogation information to generate individualized risk estimates.^9,10^ However, the contribution of established arrhythmic markers such as non-sustained ventricular tachycardia (NSVT) to machine-learning model performance remains incompletely understood. Moreover, many reported models lack geographic external validation and often do not account for real-world programming parameters that directly influence therapy delivery.

We therefore developed an ML model to predict appropriate ICD therapy using routinely available clinical, ECG-derived, device interrogation, and device programming parameters. The model is intended for post-implant dynamic risk stratification during routine ICD follow-up, not for pre-implant ICD decision-making. By integrating baseline characteristics with updated ECG and ICD interrogation/programming parameters, it aims to identify patients at increased risk of appropriate ICD therapy and support timely optimization of preventive management. We evaluated a base model without explicit arrhythmic burden information and quantified the incremental value of NSVT. Finally, we performed geographic external validation in an independent cohort from a third hospital site.

## Methods

This study was performed in accordance with the EHRA AI checklist for studies on artificial intelligence in cardiac arrhythmia care (see Supplementary material online, *Table S2*).^11^

### Study population and outcome

We retrospectively analysed consecutive adult patients (age ≥ 18 years) who underwent ICD implantation between 2020 and 2025 at two hospital sites of the German Heart Center of the Charité in Berlin (development cohort). To ensure clinical relevance, the analysis was restricted to this contemporary period, as advances in heart failure management may have altered the incidence and predictors of ventricular arrhythmias and ICD therapies; therefore, older cohorts were not included to increase sample size. An independent cohort from a third hospital site was assembled for geographic external validation using identical inclusion and exclusion criteria. The inclusion criteria were patients aged ≥ 18 years with an indication for ICD therapy of Class I or II according to current ESC guidelines. Patients with suspected inappropriate ICD therapy were excluded if the corresponding intracardiac electrograms were unavailable. We performed a prediction-model-specific sample-size assessment using the Riley framework.^12^ The calculation was based on the number of candidate predictor parameters, the observed outcome prevalence, an anticipated C-statistic of 0.80, and a target global shrinkage factor of 0.90.^13^ The anticipated C-statistic of 0.80 was selected as a clinically plausible value based on previous ICD-based machine-learning studies reporting comparable AUC values.^9,14^

The index time point was ICD implantation. In addition to remote monitoring, patients underwent routine ICD device follow-up either at our tertiary centre or by their primary cardiologists. The primary endpoint was the occurrence of appropriate ICD therapy, defined as anti-tachycardia pacing or shock therapy delivered for ventricular tachyarrhythmias during follow-up through 2025. ICD therapies were classified as appropriate or inappropriate based on intracardiac electrogram recordings and adjudicated by experienced electrophysiologists. Outcome status was determined solely by the occurrence of confirmed appropriate ICD therapy; patients with both appropriate and inappropriate therapies were classified as outcome-positive, whereas confirmed inappropriate-only therapies were classified as outcome-negative. Therapy delivery occurred under real-world clinical programming; programming settings were individualized and optimized by treating physicians and could be adjusted during follow-up.

### Predictors

Candidate predictors were pre-specified and extracted from routinely available clinical data (demographics, comorbidities, heart failure characteristics, and indication for ICD implantation), echocardiography (LVEF), ECG-derived measures (conduction intervals and waveform gradients), device interrogation measures (lead impedance, pacing thresholds, and shock coil impedance), and device programming parameters (detection interval/cycle length, number of intervals to detect, and number of programmed detection zones). ECG-derived and device-derived parameters were extracted from measurements obtained immediately prior to the arrhythmic event in patients who experienced the endpoint, or from the most recent available assessment in patients without an endpoint. Device programming parameters were extracted at the time of ICD implantation. No automated feature extraction or dimensionality reduction techniques were applied. No univariable screening was performed before model training; all pre-specified candidate predictors were entered into the modelling pipeline without selection based on univariable associations.

NSVT was defined according to the timing of ascertainment. Before ICD implantation, NSVT was defined as ≥ 3 consecutive ventricular beats at a rate of ≥ 120 b.p.m. terminating spontaneously within 30 s. After ICD implantation, device-detected NSVT was defined as a ventricular tachyarrhythmia with a programmed detection interval of < 500 ms that terminated spontaneously before delivery of ICD therapy. NSVT was ascertained from ICD-detected episodes or Holter ECG documented prior to the outcome event or, in patients without events, prior to the last available follow-up, excluding episodes occurring within 30 days before appropriate ICD therapy, and was included as a binary predictor in the extended model. To quantify its incremental value, we defined two model specifications: (i) a base model excluding NSVT and (ii) an extended model including NSVT, while keeping the modelling pipeline identical.

### Preprocessing and missing data

All preprocessing was implemented using scikit-learn pipelines and fitted strictly within cross-validation folds to prevent data leakage. Continuous variables were robustly coerced to numeric values and imputed using the median. Categorical variables were imputed using the most frequent category and one-hot encoded. No explicit missingness indicators were included to avoid learning centre-specific missingness patterns. In addition, all data splitting was performed at the patient level, ensuring that no individual contributed data to both training and validation sets. Furthermore, no data augmentation or reuse of individual samples was performed. Time-updated variables, including NSVT episodes during follow-up, were incorporated as predictors, reflecting dynamic risk assessment while avoiding unintended data leakage. The outcome classes were imbalanced, reflecting the real-world distribution of appropriate ICD therapy. No explicit resampling or rebalancing techniques were applied.

### Model development, internal validation, and external validation

Model development used nested stratified cross-validation in the development cohort (5 outer folds for unbiased performance estimation; 3 inner folds for hyperparameter tuning by grid search). Candidate algorithms included logistic regression, random forest, extremely randomized trees, and histogram-based gradient boosting classifiers. Model selection was based on the mean out-of-fold receiver operating characteristic area under the curve [receiver operating characteristic (ROC)-area under the curve (AUC)] across outer folds.

Discrimination was assessed using ROC-AUC and precision–recall AUC (average precision). Calibration was assessed using calibration curves, Brier score, and calibration slope/intercept. Threshold-dependent performance was summarized at a fixed probability threshold of 0.50 and at a Youden index–optimized threshold derived from out-of-fold predictions. This development-derived threshold was fixed before application to the external validation cohort, and no threshold optimization was performed in the external validation cohort.

For external validation, the final refitted model (trained on the full development cohort with the selected hyperparameters) was applied to the independent validation cohort without further tuning or recalibration. Uncertainty for key metrics was estimated using nonparametric bootstrap resampling (2000 iterations).

The study population included a broad range of patients with respect to age and sex. Model development and validation were performed across these groups to support generalizability.

## Ethics

The study was conducted in accordance with the Declaration of Helsinki and approved by the institutional ethics committee (approval number EA1/180/25). Given the retrospective design and pseudonymized data analysis, the requirement for informed consent was waived. Analyses were performed for research purposes only and had no influence on clinical management. This study corresponds to a TRIPOD Type 3 prediction model development, internal validation, and geographic external validation study. The machine learning model developed in this study is intended for research purposes only and has not been approved by any regulatory authority.

## Results

### Study population

Two patients without appropriate ICD therapy had possible inappropriate ICD therapy documented in the electronic medical records; however, intracardiac electrograms were unavailable for adjudication, and these patients were therefore excluded from the analysis. The development cohort comprised 514 ICD recipients, of whom 77 (15%) experienced appropriate ICD therapy during follow-up (mean follow-up 404 ± 510 days). The development cohort included 514 patients and 77 outcome events, corresponding to an events-per-predictor-parameter value of ∼ 2.5. The prediction-model-specific sample-size assessment using the Riley framework estimated a minimum sample size of 1697 patients and 255 events for model development. Baseline characteristics are shown in *Table 1*, and ECG/device interrogation characteristics in *Table 2*. The intervals from ECG or ICD interrogation/device assessment to appropriate ICD therapy or the last available follow-up are summarized in the Supplementary material online, Table. ECG-derived parameters showed similar intervals between groups, whereas device-derived parameters showed longer intervals in patients with appropriate ICD therapy. Ventricular tachycardia/ventricular fibrillation, listed as an underlying disease in *Table 1*, was defined as ventricular arrhythmias with preserved LVEF and primarily represents patients undergoing secondary prevention. All parameters listed in *Tables 1* and *2* were used to build the ML model. There were no significant differences in ECG- and device-derived parameters between patients with and without ICD therapy. The detection interval in the ICD therapy group was significantly longer than that in the non-ICD therapy group. The external validation cohort included 220 ICD recipients, of whom 51 (23%) experienced appropriate ICD therapy; baseline characteristics are summarized in *Table 1*.

**Table 1:** Patients characteristics

Model development cohort	Total *n* = 514	ICD therapy (−) *n* = 437	ICD therapy (+) *n* = 77	*P* value
Male, *n* (%)	407 (79.2)	342 (78.3)	65 (84.4)	0.29
Age, years	60.1 ± 14.2	60.3 ± 13.9	58.7 ± 15.9	0.36
BMI, kg/m^2^	27.5 ± 9.84	27.4 ± 10.2	28.2 ± 7.22	0.51
Secondary prophylaxis, *n* (%)	209 (40.8)	173 (39.8)	36 (46.8)	0.26
ICM, *n* (%)	165 (32.3)	142 (32.7)	23 (29.9)	0.69
HCM, *n* (%)	40 (7.8)	35 (8.0)	5 (6.5)	0.82
DCM, *n* (%)	120 (23.3)	102 (23.4)	18 (23.4)	1.00
VT/VF, *n* (%)	114 (22.3)	97 (22.3)	17 (22.1)	1.00
Sarcoidosis, *n* (%)	16 (3.1)	12 (2.8)	4 (5.2)	0.28
NSVT, *n* (%)	132 (25.7)	74 (16.9)	58 (76.3)	<0.01
Βeta blocker, *n* (%)	368 (72.4)	318 (73.4)	52 (67.5)	0.49
Class 3 antiarrhythmic drug, *n* (%)	42 (8.3)	32 (7.4)	10 (13.2)	0.11
Hypertension, *n* (%)	249 (48.7)	215 (49.4)	34 (44.7)	0.46
Diabetes, *n* (%)	120 (23.5)	105 (24.1)	15 (19.7)	0.47
Creatinine, mg/dL	1.23 ± 1.10	1.25 ± 1.22	1.12 ± 0.455	0.17
Atrial fibrillation, *n* (%)	143 (28.0)	119 (27.4)	24 (31.6)	0.9
Inappropriate ICD therapy, *n* (%)	18 (3.5)	0 (0.0)	18 (23.4)	<0.01
LVEF, %	42.9 ± 14.7	42.4 ± 15.5	44.4 ± 11.8	0.36

Validation cohort	Total *n* = 220	ICD therapy (−) *n* = 169	ICD therapy (+) *n* = 51	*P* value
Male, *n* (%)	179 (81.4)	137 (81.1)	42 (82.4)	1.00
Age, years	66.8 ± 12.3	67.6 ± 11.8	64.5 ± 13.9	0.16
BMI, kg/m^2^	26.8 ± 4.70	27.0 ± 4.92	26.5 ± 3.94	0.50
Secondary prophylaxis, *n* (%)	128 (58.2)	84 (49.7)	44 (86.3)	<0.01
ICM, *n* (%)	51 (23.2)	45 (26.6)	6 (11.8)	0.04
HCM, *n* (%)	8 (3.6)	8 (4.7)	0 (0.0)	0.06
DCM, *n* (%)	32 (14.5)	29 (17.2)	3 (5.9)	0.07
VT/VF, *n*(%)	120 (54.5)	79 (46.7)	41 (80.4)	<0.01
Sarcoidosis, *n* (%)	2 (0.9)	1 (0.6)	1 (2.0)	0.41
NSVT, *n* (%)	89 (41.0)	48 (28.9)	41 (80.4)	<0.01
Βeta blocker, *n* (%)	156 (70.9)	121 (71.6)	35 (68.6)	0.82
Class 3 antiarrhythmic drug, *n* (%)	15 (6.8)	11 (6.5)	4 (7.8)	0.75
Hypertension, *n* (%)	139 (63.2)	108 (63.9)	31 (60.8)	0.81
Diabetes, *n* (%)	56 (25.5)	46 (27.2)	10 (19.6)	0.36
Creatinine, mg/dL	1.21 ± 0.	1.25 ± 0.85	1.09 ± 0.47	0.08
Atrial fibrillation, *n* (%)	83 (37.7)	61 (36.1)	22 (43.1)	0.46
Inappropriate ICD therapy, *n* (%)	10 (4.5)	2 (1.2)	8 (15.7)	<0.01
LVEF, %	41.8 ± 13.9	42.2 ± 14.8	40.8 ± 11.7	0.69

Data are presented as the mean ± standard deviation or *n* (%). Inappropriate ICD therapy was recorded descriptively and was not included as a predictor in any model.

BMI, body mass index; DCM, dilated cardiomyopathy; HCM, hypertrophic cardiomyopathy; ICM, ischemic cardiomyopathy; NSVT, non-sustained VT; LVEF, left ventricular ejection fraction; VT/VF, ventricular tachycardia/ventricular fibrillation.

**Table 2:** Electrocardiogram, device-derived parameters and ICD programming settings

	Total *n* = 514 [Missing data *n* (%)]	ICD therapy (−) *n* = 437 [Missing data *n* (%)]	ICD therapy (+) *n* = 77 [Missing data *n* (%)]	*P* value
ECG parameters				
Heart rate, b.p.m.	72.7 ± 15 [110 (21.4)]	72.7 ± 15 [100 (22.9)]	72.7 ± 15.2 [10 (13.0)]	0.98
PQ duration, ms	179 ± 42 [158 (30.7)]	180 ± 43.7 [140 (32.0)]	176 ± 31.9 [18 (23.4)]	0.34
P duration, ms	117 ± 19.6 [231 (44.9)]	117 ± 20 [206 (47.1)]	116 ± 17.8 [25 (32.5)]	0.58
QRS duration, ms	116 ± 27.1 [109 (21.2)]	115 ± 26 [100 (22.9)]	121 ± 31.7 [9 (11.7)]	0.13
QT duration, ms	419 ± 46.3 [109 (21.2)]	417 ± 42.2 [100 (22.9)]	425 ± 62.7 [9 (11.7)]	0.32
QTC duration, ms	455 ± 40.9 [109 (21.2)]	453 ± 37.1 [100 (22.9)]	462 ± 56 [9 (11.7)]	0.23
RR interval, ms	873 ± 194 [225 (43.8)]	877 ± 202 [208 (47.6)]	855 ± 157 [17 (22.1)]	0.37
PP interval, ms	861 ± 226 [310 (60.3)]	867 ± 230 [268 (61.3)]	834 ± 209 [42 (54.5)]	0.40
P gradient, µV/ms	53.4 ± 26.5 [156 (30.4)]	54.4 ± 26.8 [138 (31.6)]	48.6 ± 24.3 [18 (23.4)]	0.11
QRS gradient, µV/ms	34.3 ± 81.7 [110 (21.4)]	34.2 ± 82.8 [101 (23.1)]	34.5 ± 76.6 [9 (11.7)]	0.98
T gradient, µV/ms	64.3 ± 77.3 [110 (21.4)]	62.9 ± 74.6 [101 (23.1)]	71.6 ± 90.1 [9 (11.7)]	0.46
**Device-derived parameters**				
Ventricular lead impedance, Ω	505 ± 174 [158 (30.7)]	508 ± 185 [136 (31.1)]	487 ± 99.8 [22 (28.6)]	0.23
Shock coil impedance, Ω	68.5 ± 12 [269 (52.3)]	68.8 ± 12.5 [244 (55.8)]	67.6 ± 9.72 [25 (32.5)]	0.48
Ventricular threshold, V	0.42 ± 0.089 [223 (43.4)]	0.422 ± 0.097 [199 (45.5)]	0.411 ± 0.032 [24 (31.2)]	0.16
ICD programming settings				
Number of detection zones	2.71 ± 0.62 [0 (0.0)]	2.72 ± 0.62 [0 (0.0)]	2.70 ± 0.59 [0 (0.0)]	0.85
Detection interval, ms	332 ± 33.4 [122 (23.7)]	330 ± 32.2 [117 (26.8)]	343 ± 36.9 [5 (6.5)]	<0.01
Number of intervals to detection	40.8 ± 18.4 [210 (40.9)]	40.8 ± 18.5 [190 (43.5)]	40.8 ± 18.2 [20 (26.0)]	0.99

Data are presented as the mean ± standard deviation or *n* (%).

### Base model excluding non-sustained ventricular tachycardia

**Figure 1:**
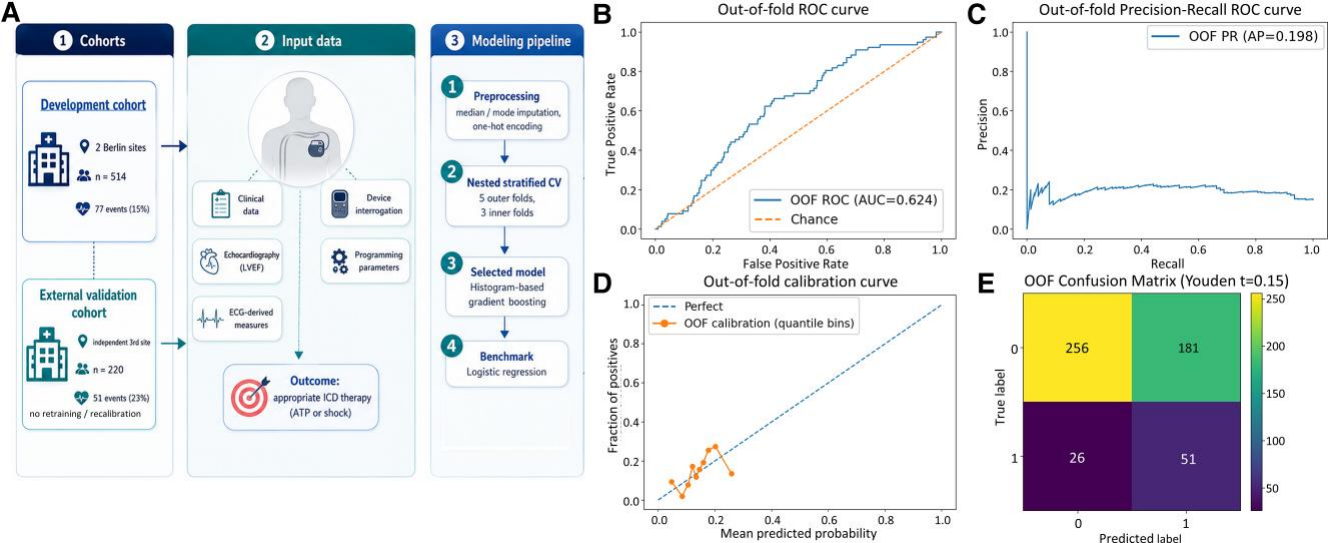
Model development workflow and performance of the base model excluding NSVT. (*A*) Overview of the study cohorts, input data sources, and modelling pipeline for the base model. The development cohort included 514 patients from two Berlin sites, of whom 77 experienced appropriate ICD therapy; the external validation cohort included 220 patients from an independent third site, of whom 51 experienced appropriate ICD therapy. Input data included clinical variables, echocardiography-derived LVEF, ECG-derived measures, device interrogation data, and programming parameters. (*B*) Out-of-fold receiver operating characteristic curve in the development cohort. (*C*) Out-of-fold precision–recall curve in the development cohort. (*D*) Out-of-fold calibration curve using quantile bins, showing agreement between predicted and observed event probabilities for the base model. (*E*) Out-of-fold confusion matrix at the Youden index–optimized threshold of 0.15, demonstrating moderate sensitivity but limited specificity in the absence of NSVT information.

As a conventional statistical benchmark for the base-model feature set, excluding NSVT, logistic regression achieved a mean out-of-fold ROC-AUC of 0.510, an average precision of 0.200, and a Brier score of 0.193. The selected histogram-based gradient boosting model showed higher discrimination (out-of-fold ROC-AUC 0.624; average precision 0.198; *Figure 1*) and improved probabilistic accuracy (Brier score 0.133). At the Youden index–optimized threshold, sensitivity was 0.66 and specificity was 0.59, indicating limited standalone performance when arrhythmic burden information was not explicitly available.

**Figure 2:**
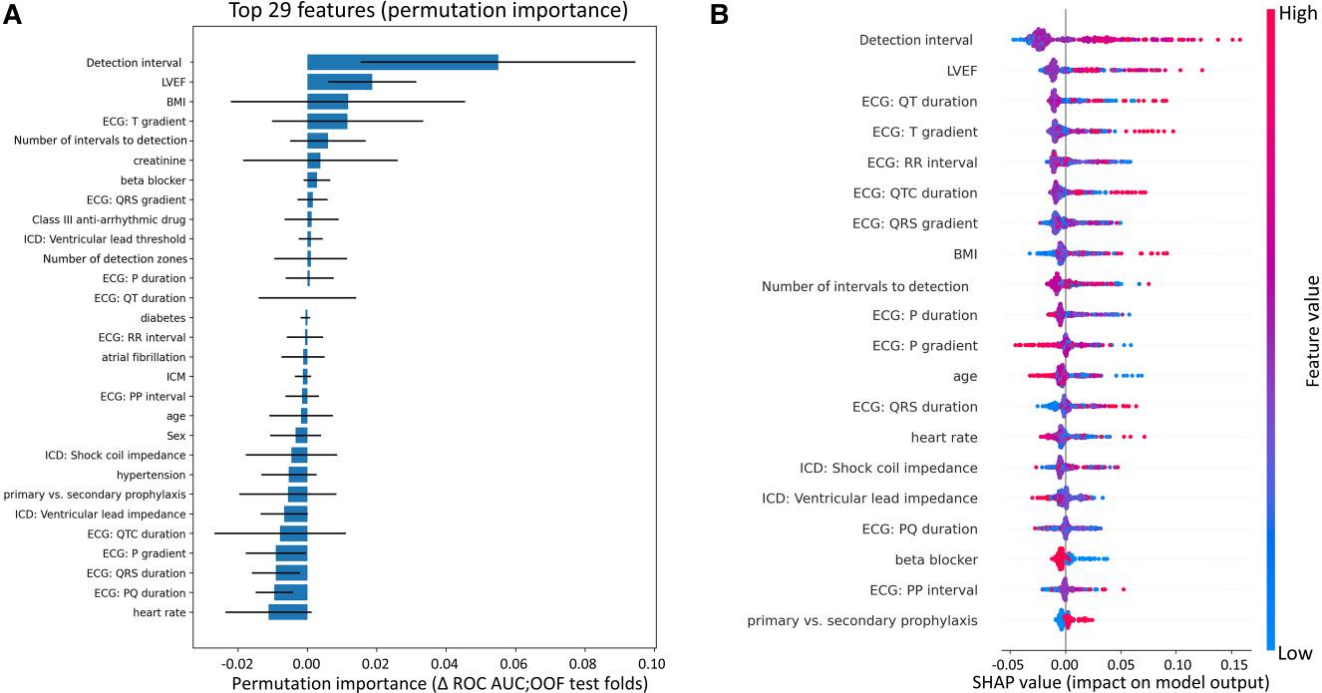
Model interpretation excluding NSVT (base model). (*A*) Permutation importance computed on held-out outer folds (change in ROC-AUC following feature permutation) and (*B*) SHAP summary plot for the base model excluding NSVT.

Permutation importance and SHAP analyses of the base model highlighted ICD programming parameters, particularly the programmed detection interval, as key contributors, alongside LVEF and selected ECG-derived measures (*Figure 2*).

### Extended model including non-sustained ventricular tachycardia

**Figure 3:**
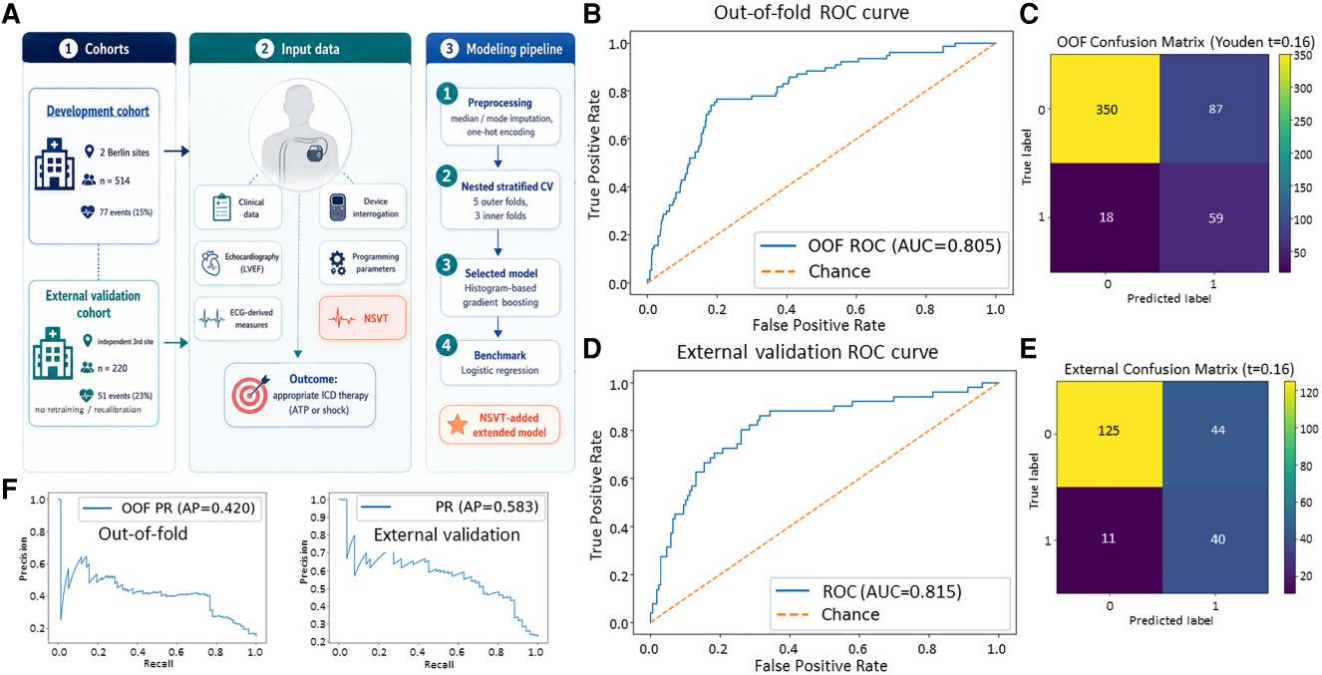
Model development workflow and discrimination of the extended model, including non-sustained ventricular tachycardia (NSVT). (*A*) Overview of the study cohorts, input data sources, and modelling pipeline for the extended model, including NSVT. Compared with the base model, NSVT was added as an additional arrhythmic burden marker while the remaining modelling pipeline was kept unchanged. (*B*) Out-of-fold receiver operating characteristic curve in the development cohort. (*C*) Out-of-fold confusion matrix in the development cohort at the Youden index–optimized threshold of 0.16. (*D*) Receiver operating characteristic curve in the external validation cohort, demonstrating preserved discrimination with ROC-AUC 0.815 without retraining or recalibration. (*E*) Confusion matrix in the external validation cohort at the pre-specified threshold (sensitivity 0.78 and specificity 0.74). (*F*) Precision–recall curves for the extended model, showing average precision of 0.420 in out-of-fold development predictions and 0.583 in the external validation cohort.

**Figure 4:**
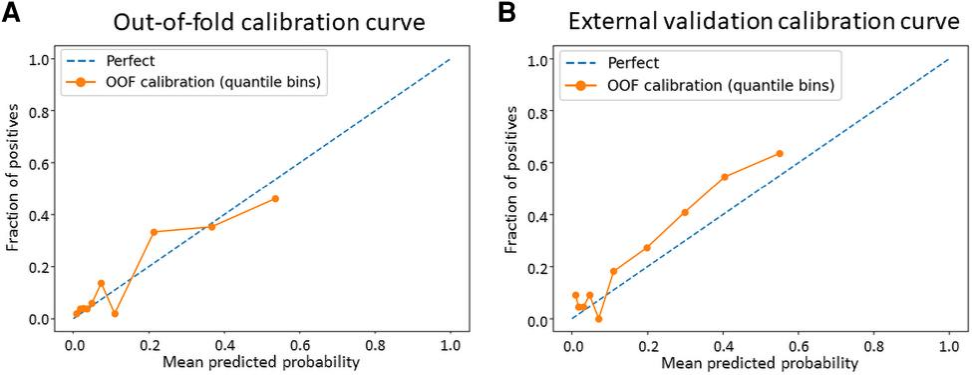
Calibration including NSVT (extended model). Calibration curves (quantile bins) for the NSVT-inclusive model in (*A*) out-of-fold development cohort predictions and (*B*) external validation.

Adding NSVT substantially improved the prediction of appropriate ICD therapy in the development cohort. As a conventional statistical benchmark, logistic regression including NSVT achieved a mean out-of-fold ROC-AUC of 0.78, average precision of 0.43 and Brier score of 0.14. Based on concatenated out-of-fold predictions, the selected histogram-based gradient boosting model achieved an ROC-AUC of 0.81 (95% CI 0.75–0.86) and an average precision of 0.42 (95% CI 0.32–0.54; *Figure 3A* and *B*). Thus, logistic regression with NSVT achieved discrimination close to that of the histogram-based gradient boosting model, suggesting that the additional gain of histogram-based gradient boosting beyond logistic regression was modest. Probabilistic performance was stable (Brier score 0.106, 95% CI 0.087–0.123) with moderate calibration (*Figure 4A*).

At a fixed probability threshold of 0.50, sensitivity was 0.19, and specificity was 0.96. Using a Youden index–optimized threshold derived from out-of-fold predictions, sensitivity increased to 0.73 with specificity 0.80 (balanced accuracy 0.77; positive predictive value 0.40; *Figure 3C*). Geographic external validation demonstrated preserved discrimination (ROC-AUC 0.815, 95% CI 0.739–0.884; average precision 0.583, 95% CI 0.452–0.727; *Figure 3D* and *E*) and acceptable calibration (*Figure 4B*; Brier score 0.137).

Model performance in the training data was comparable to cross-validation results, indicating no evidence of substantial overfitting (see Supplementary results). Misclassifications were observed in both false-positive and false-negative cases, particularly in patients without NSVT, highlighting the challenges of risk prediction in the absence of clear arrhythmic risk markers.

In external validation, threshold-dependent performance at 0.50 yielded sensitivity 0.20 and specificity 0.97. At a pre-specified lower threshold of 0.16, sensitivity increased to 0.78 with specificity 0.74 (positive predictive value 0.48; negative predictive value 0.92; *Figure 3F*).

**Figure 5:**
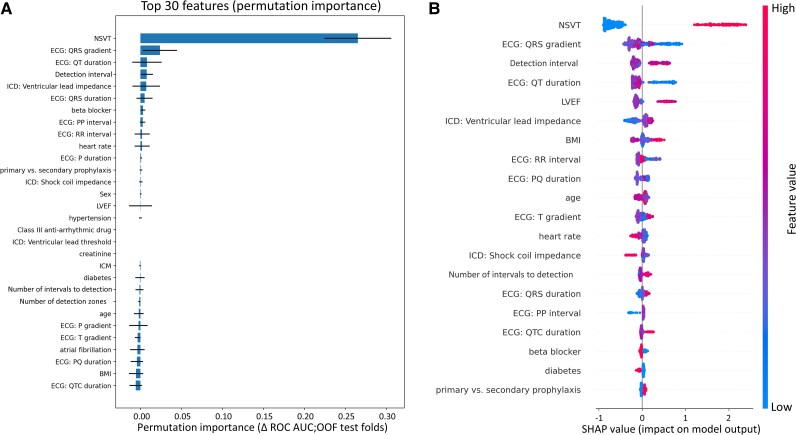
Model interpretation including NSVT (extended model). (*A*) Permutation importance and (*B*) SHAP summary plot for the NSVT-inclusive model. Permutation importance is computed on held-out outer folds to avoid data leakage; SHAP values are computed for the final refitted model.

Consistent with the benchmark comparison, permutation importance and SHAP analyses of the extended model identified NSVT as the dominant contributor to discrimination, followed by ECG-derived measures (e.g. QRS gradient, QT duration) and selected interrogation/programming parameters (e.g. detection interval and lead impedance) with smaller incremental contributions (*Figure 5*).

Decision curve analysis in the external validation cohort suggested a modest, threshold-dependent net benefit compared with treat-all and treat-none strategies across clinically plausible threshold probabilities (see Supplementary material online, *Figure S1*).

### Subgroup analyses

In patients undergoing ICD implantation for primary prophylaxis (*n* = 303), discrimination remained good (out-of-fold ROC-AUC 0.805; Supplementary material online, *Figure S2*). In secondary prophylaxis patients (*n* = 209), discrimination was slightly higher (out-of-fold ROC-AUC 0.831; Supplementary material online, *Figure S3*). Across both subgroups, NSVT remained the most influential predictor in permutation importance analyses. In an exploratory subgroup analysis restricted to patients with ischemic cardiomyopathy (see Supplementary material online, *Figure S4*), external validation showed moderate discrimination (ROC-AUC 0.767, 95% CI 0.613–0.910; average precision 0.364, 95% CI 0.096–0.687) with acceptable overall probabilistic accuracy (Brier score 0.095, 95% CI 0.051–0.149). At the pre-specified threshold of 0.19, sensitivity was 0.83, specificity 0.67, positive predictive value 0.25, and negative predictive value 0.97.

### Sensitivity analysis

In a baseline-restricted sensitivity analysis, NSVT and all other predictors were limited to measurements obtained within 90 days after ICD implantation. The model showed attenuated but still above-chance discrimination, with an out-of-fold ROC-AUC of 0.679, average precision of 0.256, Brier score of 0.124, and calibration slope of 0.969. At the development-derived Youden threshold of 0.156, sensitivity was 75.3%, with specificity 54.2%, PPV 22.5%, NPV 92.6%, and balanced accuracy 64.8%. Early NSVT remained the dominant predictor, followed by LVEF, device parameters, and ECG-derived measures, indicating that the predictive signal persisted under baseline restriction but was weaker than in the time-updated model.

To assess the robustness of model performance and feature attribution in the presence of substantial missingness, we also performed a sensitivity analysis excluding variables with > 40% missingness. The reduced model excluded P duration, RR interval, PP interval, shock coil impedance, ventricular threshold, and the number of intervals to detection. The results were broadly consistent with the main analysis. A nested cross-validated histogram-based gradient boosting classifier achieved good out-of-fold discrimination (ROC-AUC 0.799, PR-AUC 0.415) and a Brier score of 0.106; the calibration slope of 0.751 suggested some overfitting or overly dispersed risk estimates. At the default 0.50 threshold, performance was highly specific but insensitive (sensitivity 16.9%, specificity 97.3%, PPV 52.0%, and NPV 86.9%), whereas the Youden threshold of 0.125 shifted the model towards screening performance (sensitivity 75.3%, specificity 76.7%, PPV 36.3%, and NPV 94.6%). Model interpretability was dominated by NSVT, followed by ECG-derived depolarization/repolarization metrics, detection interval, LVEF, ICD lead impedance, BMI, and heart rate. This feature hierarchy was clinically plausible, but the dominant contribution of NSVT should be acknowledged as both the main predictive signal and a potential source of model dependence.

## Discussion

In this retrospective multi-site study, we developed an ML model for the prediction of appropriate ICD therapy using routinely available clinical, ECG-derived, device interrogation, and device programming parameters, and performed geographic external validation in an independent cohort. The extended model, including NSVT, achieved good discrimination and acceptable calibration under strict out-of-fold evaluation and preserved these properties on external validation without model updating. NSVT accounted for most of the improvement in predictive performance across modelling approaches in a real-world clinical setting. Notably, logistic regression, including NSVT, achieved discrimination similar to that of the histogram-based gradient boosting model, suggesting that much of the predictive gain was driven by NSVT rather than model complexity alone. These findings suggest potential transportability of our approach across the studied sites, although a prospective multicentre evaluation is needed to confirm clinical utility. The external validation cohort differed clinically from the development cohort, with a higher proportion of secondary-prevention patients, higher NSVT prevalence, and a higher event rate. While such heterogeneity is an important aspect of geographic external validation, these case-mix differences should be considered when interpreting the preserved external discrimination. In particular, the higher prevalence of secondary-prevention indication and NSVT may have contributed to a clearer risk gradient in the validation cohort. In addition, the number of outcome events in the external validation cohort was limited. Consequently, the external AUROC and especially the average precision should be interpreted with caution because the precision of these estimates is constrained by the borderline event count. Further validation in larger and more diverse independent ICD cohorts would provide more precise estimates of model performance. The central role of NSVT in our model is consistent with prior reports linking post-implantation NSVT to subsequent ICD therapies. Zhou *et al*. and Makimoto *et al*. reported that NSVT detected by the ICD after implantation was associated with subsequent ICD therapies in prophylactic ICD patients.^15,16^ In contrast, three previous studies demonstrated that NSVT identified prior to ICD implantation was not associated with ventricular arrhythmic events in the prophylactic ICD population.^17–19^ In the present study, we evaluated NSVT both before and after ICD implantation. Therefore, our findings highlight that the prognostic value of NSVT depends strongly on how and when it is ascertained, and that arrhythmic burden markers remain central to the prediction of appropriate ICD therapy within modern modelling frameworks.

The observed rate of appropriate ICD therapy in our cohort (15% during a mean follow-up period of 404 days) was within the range reported in prior studies, supporting the representativeness of the study population.^20–22^ Model performance should be interpreted within the appropriate clinical context. A recent study using longitudinal ECG and clinical data with dynamic machine-learning approaches reported modest discrimination for predicting ICD therapies (AUC ∼ 0.74).^23^ Recent multimodal AI studies incorporating cardiac magnetic resonance imaging have reported AUCs of ∼ 0.89 and 0.81 in internal and external cohorts, respectively,^24^ but these approaches rely on advanced imaging features not routinely available in clinical practice. In contrast, our model, based on routinely available clinical and device-related variables, achieved good external discrimination (AUC 0.82), although this performance was largely driven by NSVT. Integration of imaging-based substrate assessment and arrhythmic burden markers, such as NSVT, may further improve the prediction of appropriate ICD therapy. Importantly, our study further extends prior work by explicitly incorporating ICD programming parameters, which are known to influence therapy delivery but are often not accounted for in previous models.

Although both sensitivity and specificity are clinically important metrics for models predicting serious clinical outcomes, avoiding missed events is particularly important given the severity of the outcome in this study. Therefore, high sensitivity and negative predictive value may need to be prioritized in this setting, while lower specificity and positive predictive value should be interpreted in this clinical context. In the validation cohort, the model showed a relatively low specificity of 0.74 and a positive predictive value of 0.48, which was comparable to that of a previous ML-based study predicting appropriate ICD activation (PPV, 0.38).^25^ This performance appears clinically reasonable for a model predicting appropriate ICD therapy.

Device-derived parameters, such as lead impedance and sensing metrics, were deliberately included to capture aspects of electrical stability, lead performance, and device—myocardium interaction that are not reflected in standard clinical variables. This is consistent with a previous study by Cha *et al*., who demonstrated that machine learning applied to ICD-acquired intracardiac electrograms could predict ventricular arrhythmias seconds before their onset, achieving an AUC of 0.83,^9^ thereby suggesting the presence of dynamic electrophysiological changes immediately preceding arrhythmia initiation. However, in the present study, we focused on routinely available ICD-derived measurements, including ventricular pacing thresholds, shock coil impedance, and lead impedance. Feature importance and SHAP-based analyses consistently indicated that these parameters contributed incrementally to overall model performance. This suggests that while device parameters may reflect downstream manifestations of disease severity or structural substrate, when obtained at a single time-point, they provide limited independent prognostic information for arrhythmic therapy when considered alongside established clinical and arrhythmic markers. Notably, programming parameters, particularly the programmed detection interval, emerged as predictors when NSVT was excluded and remained contributory in the extended model. In real-world clinical practice, patients exhibit heterogeneous arrhythmic risk, and ICD programming settings vary accordingly. Because appropriate ICD therapy is inherently modulated by programming (rate cut-offs, detection times, and number of zones), incorporating programming variables helps contextualize therapy as an outcome under real-world device management. However, programming variables should not be interpreted causally or as directly actionable predictors. Changes in detection intervals or zones may alter the likelihood of ICD therapy without necessarily modifying the underlying arrhythmic risk, and therefore do not provide direct guidance for clinical management.

Previous studies indicated that among prophylactic ICD patients, 25–27% did not receive appropriate ICD therapies during the time frame of their first generator,^26,27^ and LVEF improves to > 35% in about 32–40% of patients after implantation,^28,29^ highlighting the heterogeneity of long-term ICD benefit. Such heterogeneity in clinical outcomes among ICD recipients may be better understood and interpreted through the application of machine learning–based approaches. Kolk *et al*. showed that a deep learning model integrating late gadolinium enhancement magnetic resonance imaging, 12-lead ECG, and clinical data can predict malignant ventricular arrhythmias before ICD implantation, outperforming single-modality approaches.^30^ Conversely, other multicentre studies have reported that multimodal machine learning models combining clinical data and ECG time-series can identify ICD recipients at high risk of non-arrhythmic mortality, thereby highlighting the utility of machine learning in identifying patients less likely to benefit from ICD therapy.^31^ From a clinical perspective, individualized risk estimates could support follow-up intensity or downstream evaluation strategies, and may be relevant when reassessing ICD benefit at generator replacement. However, prospective evaluation is required before clinical implementation, and threshold selection should be aligned with the intended clinical action and acceptable trade-offs between false positives and false negatives.

## Limitations

Several limitations merit consideration. First, the retrospective observational design limits causal inference and the model was not used to guide clinical management. Second, although geographic external validation was performed, validation was limited to a single additional site within one healthcare system and may not generalize to other institutions, device vendors, or programming practices, which may limit applicability to other populations or clinical environments. Third, appropriate ICD therapy was modelled as a binary outcome over follow-up and did not explicitly account for time-to-event or competing risks. While we acknowledge that incorporating such approaches may provide a more comprehensive assessment, this was beyond the scope of this analysis, particularly given the use of predictors defined at varying reference time points, making a uniform time-zero difficult to establish, and could be addressed in future work using survival modelling techniques. Fourth, ECG, interrogation, and programming parameters were analysed as cross-sectional measurements and therefore did not capture longitudinal changes or programming updates over time. These dynamic data may provide additional prognostic information for real-time risk prediction. Future studies using standardized follow-up protocols, longitudinal remote-monitoring datastreams, and formal landmark or time-updated prediction designs are warranted. Because predictor assessment was not anchored to a uniform landmark time point, timing-related ascertainment bias cannot be fully excluded, particularly for NSVT. Further studies using formal landmark or fully time-updated prediction designs are warranted. Fifth, although subgroup analyses by underlying conditions (e.g. hypertrophic cardiomyopathy and dilated cardiomyopathy) and analyses stratified by primary and secondary prevention would have been informative, these analyses were not performed due to the limited sample size, which may increase the risk of overfitting and unstable estimates in machine learning models. Sixth, NSVT was defined based on both pre-implant and post-implant detections, including episodes detected by ICDs. As a result, it was not feasible to analyse the relationship between the timing of NSVT relative to implantation and ICD therapy. Seventh, NSVT ascertainment was harmonized at the clinical-definition level, but device manufacturers, detection algorithms, interval-counting rules, and programmed ventricular tachycardia/ventricular fibrillation zones varied across patients. These residual differences may have influenced NSVT prevalence and model performance across sites. Eighth, the development cohort was smaller than the sample size recommended by the Riley framework for a new prediction model with this number of candidate predictor parameters. The limited number of outcome events relative to the candidate predictor set may have increased the risk of model instability and optimism. Although nested cross-validation was used to reduce optimism in performance estimation, it cannot fully compensate for limited event numbers. Therefore, the predictive performance of the model should be interpreted with caution and requires further validation in larger, contemporary, multicentre independent ICD cohorts. Ninth, patients with suspected inappropriate ICD therapy were excluded when intracardiac electrograms were unavailable for adjudication. Although this approach was chosen to ensure reliable classification of ICD therapies, it may have introduced selection bias by excluding patients with possible inappropriate-only therapy. Finally, missing data were handled by imputation within the cross-validation pipeline to avoid data leakage. However, residual bias due to missing-not-at-random mechanisms cannot be excluded, and imputation of variables with substantial missingness may have introduced uncertainty and potentially biased feature attribution, including SHAP-based interpretations.

## Conclusions

Machine-learning models integrating routinely available clinical, ECG, and ICD interrogation/programming data may enable prediction of appropriate ICD therapy, with preserved performance on geographic external validation. Comparison with logistic regression suggested that NSVT was the dominant contributor to predictive performance across modelling approaches, with only modest incremental gain from the machine-learning pipeline, while programming parameters may provide complementary information when arrhythmic burden is not explicitly available. These findings support further multicentre validation and prospective evaluation.

## Supplementary Material

ztag102_Supplementary_Data

## Data Availability

The data underlying this article contain sensitive patient information and are therefore not publicly available. The analysis code and derived artefacts can be made available from the corresponding author on reasonable request, subject to institutional and ethical approval.
