# Supplementary material for: Prediction of appropriate implantable cardioverter-defibrillator therapy using machine learning and routinely available clinical data
Source: Eur Heart J Digit Health. 2026 Jun 26;7(6):ztag102. doi: 10.1093/ehjdh/ztag102 (PMC13344847; doi:10.1093/ehjdh/ztag102)
Supplement: ztag102_Supplementary_Data [file ztag102_supplementary_data.docx]

**Supplementary Methods**

All analyses were performed using Python (version ≥3.9) and implemented with established open-source libraries, including scikit-learn for model development and evaluation, pandas and NumPy for data handling, and SHapley Additive exPlanations (SHAP) for model interpretation. The complete modeling workflow was implemented using scikit-learn’s Pipeline and ColumnTransformer abstractions to ensure reproducibility and to strictly prevent information leakage between training and validation data. Random seeds were fixed for all stochastic components to enable deterministic replication of results.

**Candidate predictors and preprocessing**

Candidate predictors were predefined prior to model training and comprised routinely available clinical variables, echocardiographic measures, electrocardiogram-derived parameters, arrhythmic markers, device-derived measurements, and ICD programming parameters (e.g., detection interval/cycle length, detection beats count/number of intervals to detect, and the number of programmed detection zones). No outcome-driven feature selection or dimensionality reduction was performed at any stage of the analysis. Continuous variables were retained on their original scale.

All preprocessing steps were performed within the cross-validation framework. Continuous variables were coerced to numeric values using robust parsing procedures and imputed using median imputation. Categorical variables were coerced to uniform string representations, imputed using the most frequent category, and encoded using one-hot encoding. Feature scaling was applied only where required by the model class. All preprocessing parameters were learned exclusively from the training data within each cross-validation split and subsequently applied to the corresponding validation data. No explicit missingness indicators were included to avoid learning centre-specific missingness patterns.

**Device-derived parameters**

Device-derived parameters, including lead impedance, sensing amplitudes, pacing thresholds, shock coil impedance, and programming parameters were extracted from the device interrogation closest in time to the arrhythmic event for patients experiencing appropriate ICD therapy. For patients without events, device parameters were obtained from the most recent available interrogation prior to database extraction. Only a single time point per patient was included; longitudinal trends and delta-based representations of device parameters were not considered in the present analysis.

**Model classes**

Four model classes were evaluated to capture different levels of model complexity and inductive bias. Logistic regression with L2 regularization served as a linear baseline model. Random forest and extremely randomized trees (ExtraTrees) classifiers were used to model nonlinear relationships and higher-order feature interactions through ensembles of decision trees. In addition, a histogram-based gradient boosting classifier was evaluated as a boosting approach optimized for tabular data. Model classes and corresponding hyperparameter grids were specified a priori to balance interpretability, flexibility, and robustness and to reflect commonly used approaches in contemporary clinical machine-learning research. Explicit missingness indicators were not included as model features to avoid reliance on data availability patterns that may not generalize across centres or clinical workflows.

**Hyperparameter tuning and model selection**

Hyperparameter optimization was performed using grid search within the inner loop of a nested cross-validation framework. Hyperparameter grids were defined prior to analysis and included regularization strength and class weighting for logistic regression, tree depth, minimum leaf size, feature subsampling, and class weighting for tree-based models, and learning rate, tree complexity, regularization, and number of boosting iterations for gradient boosting models. Model selection within each inner cross-validation loop was based on maximization of the area under the receiver operating characteristic curve (ROC-AUC).

**Nested cross-validation and out-of-fold prediction**

To obtain unbiased estimates of predictive performance, a nested cross-validation design was employed in accordance with TRIPOD-AI recommendations for prediction model development and internal validation. Stratified K-fold cross-validation was used in the outer loop to preserve the proportion of patients experiencing appropriate ICD therapy in each fold. The inner loop was used exclusively for hyperparameter tuning and model selection.

For each patient, predicted probabilities were generated only from models trained on data not including that patient, yielding out-of-fold predictions. All reported performance metrics were derived solely from these out-of-fold predictions. The final model was selected based on mean out-of-fold ROC-AUC across outer cross-validation folds and subsequently refitted on the full dataset using the optimal hyperparameters.

**Performance evaluation**

Model performance was evaluated across complementary dimensions. Discrimination was assessed using ROC-AUC and precision–recall AUC (average precision), with the latter emphasized due to class imbalance. Calibration was evaluated using the Brier score and calibration curves comparing predicted and observed event probabilities across quantile-based risk strata. Calibration slope and intercept were estimated using logistic recalibration of predicted probabilities. Variability in performance across cross-validation folds is reported as mean ± standard deviation or via bootstrap-derived confidence intervals, as appropriate.

**Threshold-dependent analyses and decision curve analysis**

For descriptive purposes, threshold-dependent performance was explored by generating confusion matrices using a Youden index–optimized probability threshold derived from out-of-fold predictions. Fixed-threshold analyses were additionally examined to illustrate sensitivity–specificity trade-offs. These threshold-based evaluations were used for interpretative purposes only and did not inform model training or selection. For external validation, thresholds were prespecified from the development cohort and were not optimized on the external validation data.

Clinical utility was explored using decision curve analysis by comparing the net benefit of the model with treat-all and treat-none strategies across a range of threshold probabilities. Decision curve analysis was performed in the external validation cohort using predicted probabilities from the locked final model (Supplementary Figure S1) and interpreted qualitatively to assess potential threshold-dependent net benefit.

To quantify the incremental contribution of arrhythmic burden, a base model was evaluated in which non-sustained ventricular tachycardia (NSVT) was excluded from the predictor set. The entire modelling pipeline, including preprocessing, hyperparameter tuning, nested cross-validation, and performance evaluation, was repeated unchanged. Performance from the base (NSVT-excluded) model was compared with the extended (NSVT-inclusive) model.

**Model interpretation**

Feature importance was quantified using permutation importance, defined as the decrease in out-of-fold ROC-AUC following random permutation of individual predictors in held-out outer test folds. This approach directly measures the marginal contribution of each feature to model discrimination under realistic validation conditions.

Model interpretability was further explored using SHapley Additive exPlanations (SHAP). SHAP values were computed exclusively for the final refitted model using a fixed post-preprocessing feature space to ensure stability of feature representations. Mean absolute SHAP values were used to summarize global feature contributions, and SHAP summary (beeswarm) plots were used to visualize the direction and magnitude of feature effects on individual risk predictions. SHAP analyses were used solely for interpretative purposes and did not influence model training, tuning, or selection.

**Reproducibility and ethical considerations**

All analyses were retrospective and observational, and model outputs were not used to guide clinical management. Models were developed for risk stratification and hypothesis generation rather than for direct clinical deployment. External validation and prospective evaluation are required prior to any clinical application.

The full modeling pipeline was implemented using modular, version-controlled source code. All random operations were seeded to ensure reproducibility. Detailed variable definitions, preprocessing steps, and hyperparameter grids are available upon reasonable request to facilitate independent replication.

**Subgroup analyses**

We performed subgroup analyses stratified by primary and secondary prophylaxis. Two patients had no available information on prophylaxis and were therefore excluded from the subgroup analyses.

**Supplementary Results**

In the training/development dataset, model development was performed with 5-fold cross-validation. Among candidate models, the selected HistGradientBoosting model showed the best training-phase performance, with a mean ROC AUC of 0.823 (SD 0.039) across folds. The corresponding mean PR AUC was 0.455 (SD 0.091) and mean Brier score was 0.105 (SD 0.010). Candidate-model mean ROC AUCs were 0.779 for Logistic Regression, 0.811 for Random Forest, 0.811 for Extra Trees, and 0.823 for HistGradientBoosting.

**Supplementary Table**

**Duration from ECG or ICD assessment to appropriate ICD therapy or last follow-up**

|  | Total | ICD therapy (-) | ICD therapy (+) | P value |
| --- | --- | --- | --- | --- |
| ECG parameters, day | 191.7±354.1 | 189.7±367.9 | 202.2±275.4 | 0.79 |
| Device-derived parameters, day | 57.4±164.0 | 40.1±138.8 | 156.9±245.0 | <0.01 |

Data are presented as the mean standard ± deviation

ECG, electrocardiogram; ICD, implantable cardioverter-defibrillator.

Intervals were calculated from the most recent ECG or ICD interrogation/device assessment to appropriate ICD therapy in event-positive patients, and to the last available follow-up in event-negative patients.

**Supplementary figures**


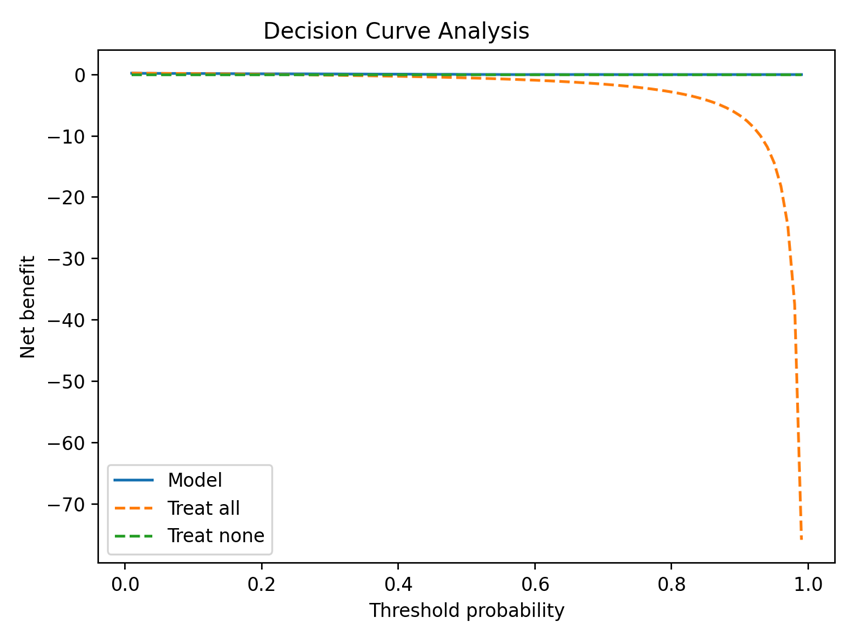


**Supplementary Figure S1. Decision curve analysis in external validation.**

External validation cohort decision curve analysis comparing the model with default strategies (“treat all” and “treat none”) across threshold probabilities.

**
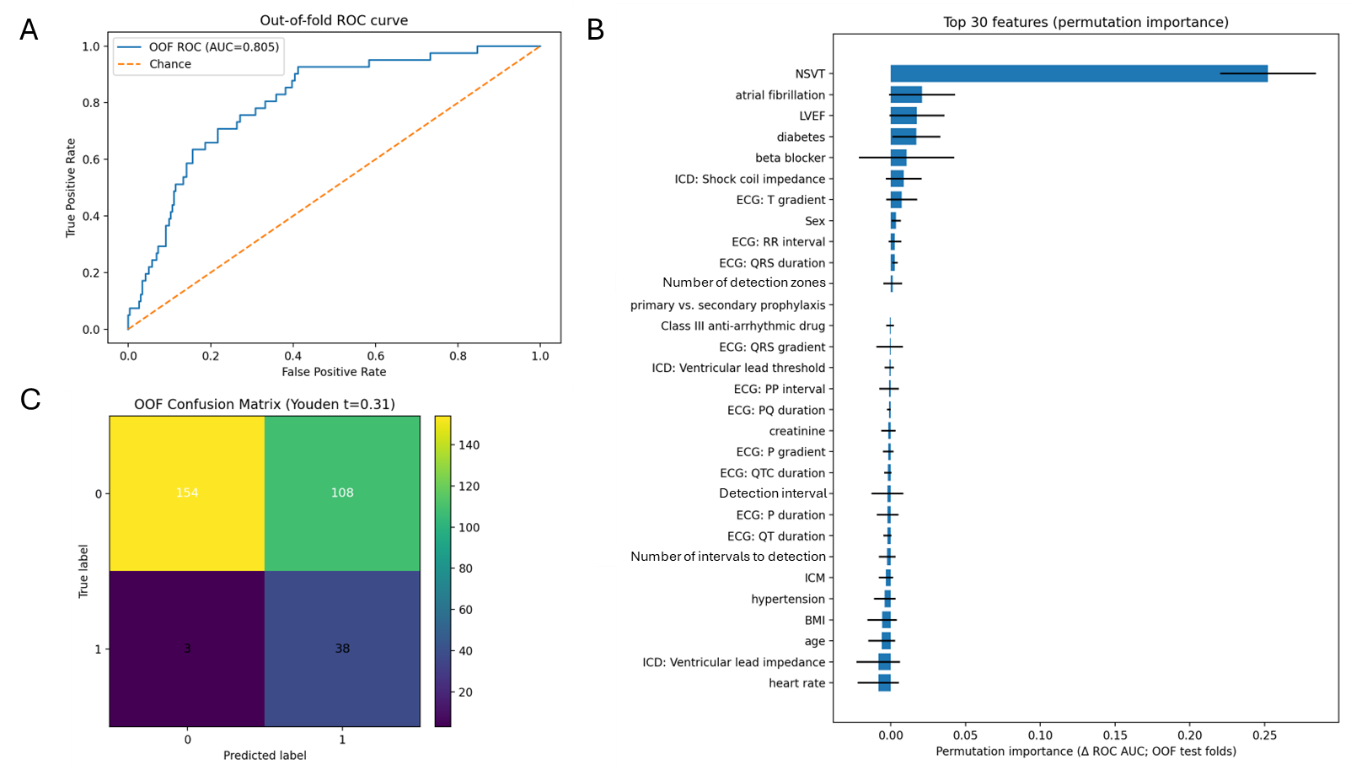
**

**Supplementary Figure S2. Model performance and feature importance in the primary prophylactic ICD subgroup.**

(A) Out-of-fold receiver operating characteristic (ROC) curve demonstrating moderate discrimination (AUC = 0.805) for prediction of appropriate ICD therapy in patients undergoing primary prophylactic ICD implantation. The dashed line indicates chance performance.

(B) Permutation feature importance analysis (mean decrease in ROC-AUC across test folds), showing non-sustained ventricular tachycardia as the dominant predictor, followed by ECG-derived markers (QT duration, QRS gradient) and selected clinical and device-related variables. Error bars indicate variability across folds.

(C) Out-of-fold confusion matrix at the Youden index–optimized probability threshold (t = 0.31).

**
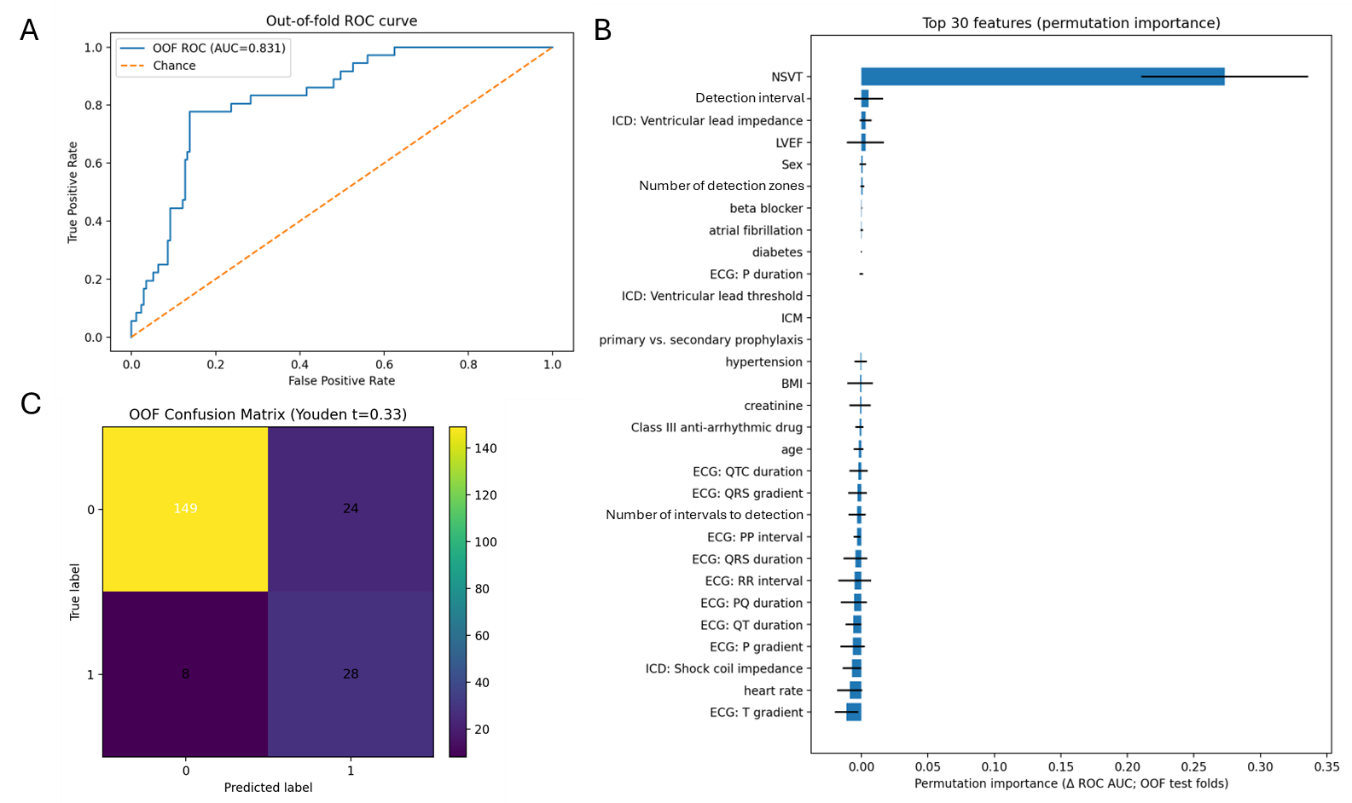
**

**Supplementary Figure S3. Model performance and feature importance in the secondary prophylactic ICD subgroup.**

(A) Out-of-fold receiver operating characteristic (ROC) curve demonstrating good discrimination for prediction of appropriate ICD therapy in patients with a secondary prophylactic ICD indication (AUC = 0.83). The dashed line indicates chance performance.

(B) Permutation feature importance analysis (mean decrease in ROC-AUC across test folds), identifying non-sustained ventricular tachycardia as the strongest predictor, followed by device and ECG-derived parameters. Error bars represent variability across folds.

(C) Out-of-fold confusion matrix at the Youden index–optimized probability threshold (t = 0.33), illustrating balanced sensitivity and specificity in this higher-risk population.

**
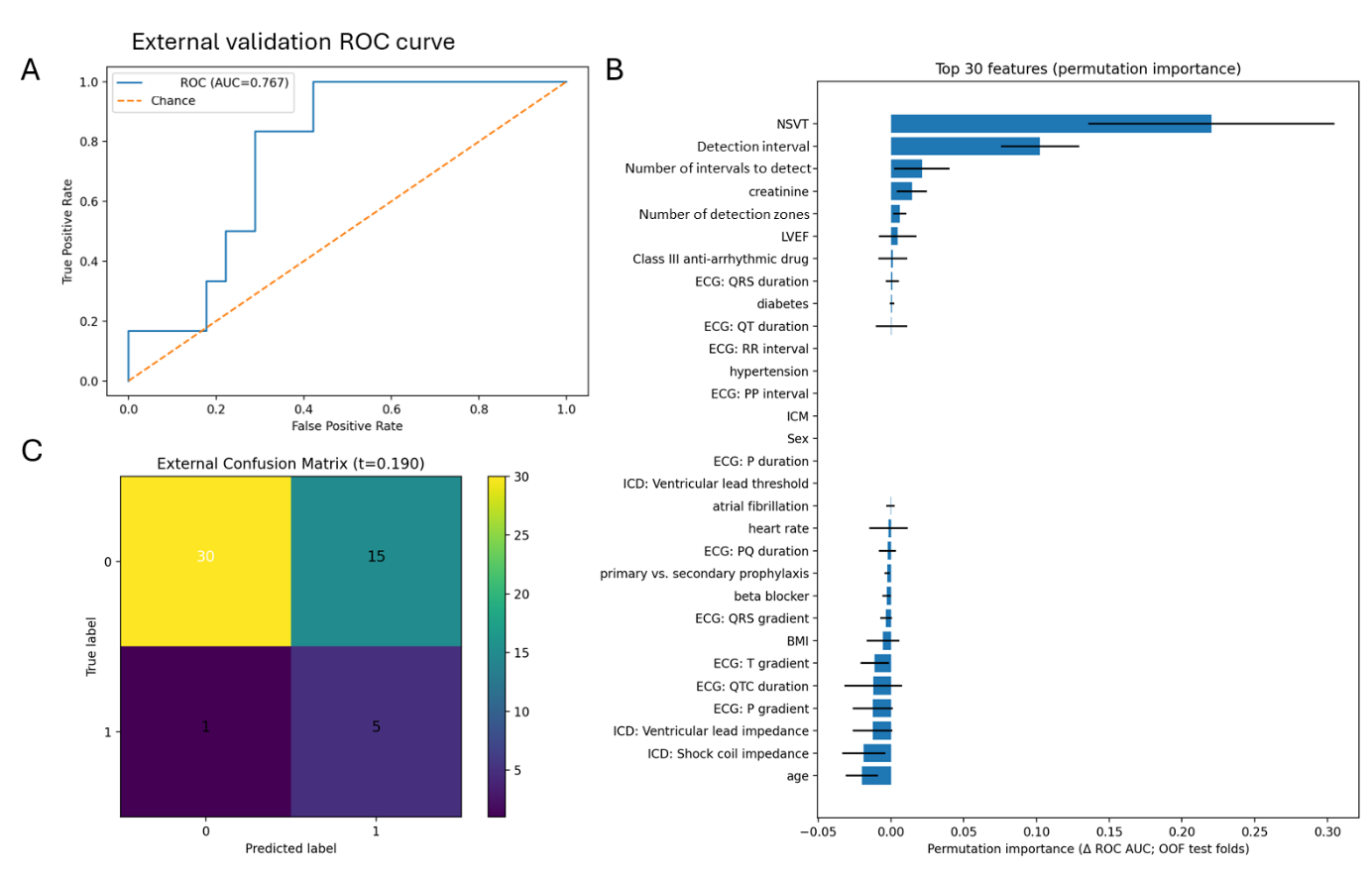
**

**Supplementary Figure S4. External validation performance and feature importance in the ischemic cardiomyopathy subgroup.** **Unlike the primary and secondary prophylaxis subgroup analyses (Supplementary Figures S2–S3), which were based on internal out-of-fold evaluation only, this subgroup analysis was performed in the geographically independent external validation cohort.**

(A) Receiver operating characteristic (ROC) curve in the external validation cohort restricted to patients with ischemic cardiomyopathy, demonstrating moderate discrimination for prediction of appropriate ICD therapy (AUC = 0.767). The dashed line indicates chance performance.

(B) Permutation feature importance analysis in the ischemic cardiomyopathy subgroup, showing non-sustained ventricular tachycardia (NSVT) as the dominant predictor, followed by ICD programming parameters and selected clinical and electrocardiographic variables. Horizontal error bars indicate variability across validation folds or resampling iterations.

(C) Confusion matrix in the external validation ischemic cardiomyopathy subgroup at the prespecified probability threshold (t = 0.190).

**Supplementary Table S1. EHRA AI checklist**

| **THE EHRA AI checklist for reporting, reading and understanding AI studies in clinical EP** | | | | |
| --- | --- | --- | --- | --- |
| **Item #** | **Category/Section** | **Explanation** | **Example from the trial** | **Page #** |
|  | **TITLE** |  |  |  |
|  | ***Title*** | Include clear terms to identify the study as using artificial intelligence, machine learning or other specific terms | *Prediction of Appropriate Implantable Cardioverter-Defibrillator Therapy Using Machine Learning and Routinely Available Clinical Data* | Title page |
|  | **INTRODUCTION** |  |  |  |
| 1 | **Intended clinical use** | Clearly describe the intended use and where in clinical workflow the model can be used and the objective of the study | The model is intended for post-implant dynamic risk stratification during routine ICD follow-up, not for pre-implant ICD decision-making. | 5 |
| 2 | **Clinical benefit** | Added benefit of AI compared to standard clinical care (gold standard) | To identify patients at increased risk of appropriate ICD therapy and support timely optimization of preventive management. | 5 |
|  | **METHODS** |  |  |  |
| 3 | **Data Collection** | Describe how data was collected | Single center (3 hospital sites), retrospective data | 5 |
| 4 | **Source (of data)** | Describe the study design or source of input data and how it was acquired | Retrospective observational study | 5 |
| 5 | **Development data set (model training data set)** | Describe the data set | Candidate predictors were prespecified and extracted from routinely available clinical data (demographics, comorbidities, heart failure characteristics, indication for ICD implantation), echocardiography (LVEF), ECG-derived measures (conduction intervals and waveform gradients), device interrogation measures (lead impedance, pacing thresholds, shock coil impedance), and device programming parameters (detection interval/cycle length, number of intervals to detect, and number of programmed detection zones). | 7, Table 1 and 2 |
| 6 | **Participants** | Describe the participants in the data sets, including eligibility criteria (inclusion and exclusion criteria). | Consecutive adult patients (age ≥18 years) who underwent ICD implantation between 2020 and 2025 at two hospital sites of the German Heart Center of the Charité in Berlin (development cohort). An independent cohort from a third hospital site was assembled for geographic external validation using identical inclusion and exclusion criteria. The inclusion criteria were patients aged 18 years or older with an indication for ICD therapy of class I or II according to current ESC guidelines. The exclusion criteria were patients with inappropriate ICD therapies when intracardiac electrograms could not be adequately reviewed. | 5-6 |
| 7 | **Comparator** | Provide clear definition of how the gold standard was collected.  Clearly describe the gold standard and ground truth including limitations. | ICD therapies were classified as appropriate or inappropriate based on intracardiac electrograms recordings and adjudicated by experienced electrophysiologists. | 6 |
| 8 | **Validation data set** | Describe the validation data set, in particular defining the data set split. | An independent cohort from a third hospital site was assembled for geographic external validation. | 6 |
| 9 | **Sample Size** | Explain how the study size was arrived at. | To ensure clinical relevance, the analysis was restricted to this contemporary period, as advances in heart failure management may have altered the incidence and predictors of ventricular arrhythmias and ICD therapies; therefore, older cohorts were not included to increase sample size. The development cohort comprised 514 ICD recipients. The external validation cohort included 220 ICD recipients. | 5, 6, 10 |
| 10 | **Outcome** | Clearly define standardized and reproducible outcome of clinical relevance. | The primary endpoint was the occurrence of appropriate ICD therapy, defined as anti-tachycardia pacing or shock therapy delivered for ventricular tachyarrhythmias. | 6 |
| 11 | **Data type (source)** | Clearly describe the data type for the study, including pre-processing | Candidate predictors were prespecified and extracted from routinely available clinical data (demographics, comorbidities, heart failure characteristics, indication for ICD implantation), echocardiography (LVEF), ECG-derived measures (conduction intervals and waveform gradients), device interrogation measures (lead impedance, pacing thresholds, shock coil impedance), and device programming parameters (detection interval/cycle length, number of intervals to detect, and number of programmed detection zones). | 7, Table 1 and 2 |
| 12 | **Data Preparation** | Input data handling, data augmentation and selection prior to analysis by the AI system. | All preprocessing was implemented using scikit-learn pipelines and fitted strictly within cross-validation folds to prevent data leakage. No explicit missingness indicators were included to avoid learning centre-specific missingness patterns. In addition, all data splitting was performed at the patient level, ensuring that no individual contributed data to both training and validation sets. Furthermore, no data augmentation or reuse of individual samples was performed. Time-updated variables, including NSVT episodes during follow-up, were incorporated as predictors, reflecting dynamic risk assessment while avoiding unintended data leakage. | 8 |
| 13 | **Balanced groups** | Clearly state how groups were balanced | The outcome classes were imbalanced, reflecting the real-world distribution of appropriate ICD therapy. No explicit resampling or rebalancing techniques were applied. | 8 |
| 14 | **Data issues (missingness / poor data / duplication / outliers)** | Describe how handling of data of poor quality/noise/missing data was performed | Continuous variables were robustly coerced to numeric values and imputed using the median. Categorical variables were imputed using the most frequent category and one-hot encoded. | 8 |
| 15 | **Feature extraction / selection / reduction** | If features are used, feature selection should be described including by whom features were extracted. | Candidate features consisted of routinely available clinical, ECG-derived, and device-related variables selected a priori based on clinical relevance and data availability. No automated feature extraction or dimensionality reduction techniques were applied. | 7-8 |
|  | **REGULATORY** |  |  |  |
| 16 | **Legal framework** | Clearly state if the software has been approved by legal authorities, e.g. Certificate of conformity (EU) or FDA approval or other, and add further details, where appropriate (e.g. risk class). | The machine learning model developed in this study is intended for research purposes only and has not been approved by any regulatory authority. | 10 |
| 17 | **Explainability** | Is the AI model explainable on the patient level or on a population level. | SHAP analysis was used to improve model interpretability. | 11-12 |
| 18 | **Ethical approval** | Provide information on ethical approval of the study. | The study was conducted in accordance with the Declaration of Helsinki and approved by the institutional ethics committee (approval number EA1/180/25). | 9 |
| 19 | **Fairness** | Describe inclusion of relevant groups in the dataset | The study population included a broad range of patients with respect to age and sex. Model development and validation were performed. across these groups to support generalizability. | 9 |
|  | **OPEN SCIENCE** |  |  |  |
| 20 | **Data availability/ Code sharing** | Is the data available on a public website? Is the code available? | The data underlying this article contain sensitive patient information and are therefore not publicly available. The analysis code and derived artefacts can be made available from the corresponding author on reasonable request, subject to institutional and ethical approval. | 22 |
| 21 | **Trial registration** | Clearly state where the trial is registered. | Not applicable, as this was a retrospective observational study and not a registered clinical trial. | - |
|  | **RESULTS** |  |  |  |
| 22 | **Participants** | Baseline demographics | Baseline characteristics are shown in Table 1 and ECG/device interrogation characteristics in Table 2. | Table 1 and 2 |
| 23 | **Training performance** | Provide results from the training data set | Development-set 5-fold cross-validation identified HistGradientBoosting as the best model, with mean ROC AUC 0.823 (SD 0.039), mean PR AUC 0.455 (SD 0.091), and mean Brier score 0.105 (SD 0.010). | SR |
| 24 | **Internal validation** | The results from the testing data set | On the cross-validated held-out testing folds, the final model achieved pooled out-of-fold ROC AUC 0.81, PR AUC 0.42, and Brier score 0.11. | 12 |
| 25 | **External validation** | The results from the external validation data set | Provided in result section using Sens, Spec and Accuracy. | 12 |
| 26 | **Model performance Internal and external validation** | Choose appropriate metric selection for reporting | Provided in result section. | 12 |
| 27 | **Performance errors** | Analysis of performance errors and how they were identified | Misclassifications were observed in both false-positive and false-negative cases, particularly in patients without NSVT, highlighting the challenges of risk prediction in the absence of clear arrhythmic risk markers. | 11,12 |
| 28 | **Performance compared to classic statistical methods** | What did the model add? | Compared with logistic regression | 11 |
| 29 | **Generalizability** | Describe the population (internal and external validation data) | Validation was limited to a single additional site within one healthcare system and may not generalize to other institutions, device vendors, or programming practices, which may limit applicability to other populations or clinical environments. | 18 |
|  | **CONCLUSION** |  |  |  |
|  | **Conclusion** | Is the conclusion supported by the dataset? | Yes - Machine-learning models integrating routinely available clinical, ECG, and ICD interrogation/programming data may enable prediction of appropriate ICD therapy, with preserved performance on geographic external validation. NSVT appears to contribute substantially to predictive performance, while programming parameters may provide complementary information when arrhythmic burden is not explicitly available. These findings support further multicenter validation and prospective evaluation. | 20 |

SR, Supplementary Results
